# Clinical characteristics and outcomes of surgical resection for brain metastases from lung adenocarcinoma

**DOI:** 10.3389/fonc.2024.1453177

**Published:** 2025-01-21

**Authors:** Ming Li, Zhiying Li, Hang Zhang, Hiroaki Wakimoto, Linlin Sun, Tiantian Wang, Shengli Zhou, Liyun Zhou

**Affiliations:** ^1^ Department of Neurosurgery, Henan Provincial People’s Hospital, People’s Hospital of Zhengzhou University, People’s Hospital of Henan University, Zhengzhou, China; ^2^ Department of Neurosurgery, The 7th People's Hospital of Zhengzhou, Zhengzhou, China; ^3^ Department of Neurosurgery, Massachusetts General Hospital, Harvard Medical School, Boston, MA, United States; ^4^ Department of Neurology, The 7th People's Hospital of Zhengzhou, Zhengzhou, China; ^5^ Department of Pathology, Henan Provincial People’s Hospital, People’s Hospital of Zhengzhou University, People’s Hospital of Henan University, Zhengzhou, China

**Keywords:** lung adenocarcinoma brain metastases, craniotomy, clinical characteristics, survival time, prognostic factor

## Abstract

**Objective:**

The purpose of this study was to explore the clinical characteristics, survival time and prognostic factors of patients undergoing craniotomy for brain metastases (BM) from lung adenocarcinoma (LUAD).

**Methods:**

A total of 208 patients with BM from LUAD who underwent craniotomy at the Zhengzhou University People’s Hospital, Henan province, China from March 2005 to October 2022 were included in this retrospective study. All patients were confirmed as BM of LUAD by histopathology. The clinical data included patient gender, age, occupation, family history of tumor, smoking history, alcohol drinking history, neurological symptoms, history of lung cancer treatment, tumor location, tumor number, tumor size, gene status, expression of S-100, CEA, Ki67, and PD-L1 by immunohistochemistry, KPS after craniotomy, whether tumor therapy was continued after craniotomy, and survival time. Univariate and multivariate Cox regression was used to analyze the prognostic factors of patients undergoing craniotomy for LUAD BM.

**Results:**

A total of 208 patients met the inclusion and exclusion criteria, including 110 males (52.9%) and 98 females (47.1%), with an average age of 61.4 years. 203 patients (97.6%) had neurological symptoms. 84 patients (40.4%) had smoking history, 89 patients (42.8%) had alcohol drinking history, and 31 patients (14.9%) had the family history of tumor. Only 5 patients (2.4%) had received lung cancer treatment before craniotomy. The intracranial location of BM was mostly in the frontal lobe (54, 26.0%) and the metastatic sites were mostly single (117, 56.3%); the metastatic tumor size was mostly between 2-5 cm (141, 67.8%). Genetically, 43.3% patients (90 cases) had EGFR mutations, and immunohistochemical analysis showed that most patients were PD-L1 positive (160, 76.9%) and Ki67 > 30% (137, 65.9%). Most patients (145, 69.7%) had KPS score under 80 after craniotomy. Only 72 patients (34.7%) received continued tumor therapy after craniotomy. 190 patients (91.3%) were successfully followed up. The median survival time was 11.5 months, and the 3-year survival rate was 15.7%. Multivariate analysis revealed that smoking history, Ki67 percentage, KPS after craniotomy, and molecular targeted therapy after craniotomy were independent factors affecting the survival time of patients.

**Conclusions:**

Although survival remains poor, patients who had no-smoking history, Ki67 percentage ≤30%, KPS≥80 after craniotomy, and molecular targeted therapy after craniotomy can improve the prognosis and prolong the survival time.

## 1 Introduction

Brain metastases (BM) are common among patients with advanced solid tumors. Estimates of the incidence of BM in the United States have varied, but approximately 200,000 new cases of BM are diagnosed in the United States every year and that 8% -10% of patients with cancer will develop BM (1). BM appear to be 10-fold more common than primary malignant brain tumors. In adults, lung, breast, skin (melanoma) and kidney are the most frequent sources of BM (2). Lung cancer, especially non-small cell lung cancer (NSCLC), is the most common primary cancer to develop BM. Approximately 50% of all patients with NSCLC metastasize to the brain (3, 4). The prognosis of patients with NSCLC BM is extremely poor. Even with the latest integrative treatment, the 5-year survival rate remains less than 5% (5).

Modern treatment for BM has dramatically changed their expected prognosis, and current available treatment options for NSCLC BM include surgery, whole brain radiotherapy (WBRT), stereotactic radiosurgery (SRS), immunotherapy and targeted therapy. However, these treatments for NSCLC BM have certain limitations. For example, although WBRT can alleviate neurological symptoms and prevent new BM (6), its dose is limited due to potential serious toxicities (such as cognitive deterioration). Patients with NSCLC BM treated with WBRT alone generally have a poor prognosis with a median survival of less than 6 months (7). SRS delivers a high dose of conformal radiation with image guidance to minimize dose to surrounding normal brain tissue, and appears to promote anti-tumor immunity that could control tumor growth and symptoms without the neurocognitive side effects of WBRT (8). However, SRS is preferred for patients with one to three BM, and was shown to not improve the overall survival of BM (9). Chemotherapy is an active treatment for BM, however, the blood-brain barrier has limited the effective penetration of many systemic therapies into the brain. Novel systemic targeted therapies in patients with driver mutations have shown impressive intracranial efficacy, however, this treatment is largely for patients who have been pre-treated with local therapies and are asymptomatic (10). Craniotomy and tumor resection is considered for patients with a single metastasis measuring 3 cm or more, those with smaller tumors such as cerebellar neoplasms associated with severe neurologic symptoms due to cerebral edema, or those with multiple tumors with advanced neurologic symptoms in whom prompt improvement of neurologic symptoms is expected from surgery (11, 12). Lung adenocarcinoma (LUAD) is the most prevalent NSCLC cancer type that accounts for 85% of lung cancer, and the brain is the main organ prone to LUAD metastasis (13, 14). However, no retrospective data suggest that surgical resection may provide better intracranial progression-free survival in LUAD BM.

To clarify the impacts of surgery of LUAD BM, we retrospectively analyzed the clinical data, survival time and prognostic factors of the patients with BM originated from LUAD who had undergone surgical resection for the brain lesions and discussed future treatment strategies for this disease.

## 2 Patients and methods

### 2.1 Patients

We retrospectively reviewed a consecutive series of patients operated for BM from the lung, treated at Zhengzhou University People’s Hospital, Henan province, China, between March 2005 and October 2022. Our inclusion criteria for craniotomy were as follows: a surgically accessible tumor location and distinct negativity for cancer in other distant regions (except primary lung cancer). The inoperability criteria included the following: the overall condition of the patient was poor and the likelihood of craniotomy resulting in severe neural complications.

In this study, the inclusion criteria included: (1) aged 18 years and older; (2) underwent craniotomy for LUAD BM; (3) histological confirmation of cancer cell origin from lung; (4) lung cancer diagnosis with functional imaging including computed tomography (CT) and magnetic resonance imaging (MRI). The exclusion criteria included: (1) Only intracranial tumor biopsy was performed, with no intracranial tumor resection; (2) Pathological or imaging data were incomplete to prove that the intracranial tumor originated from the lung; (3) With other primary intracranial tumors.

**Figure 1:**
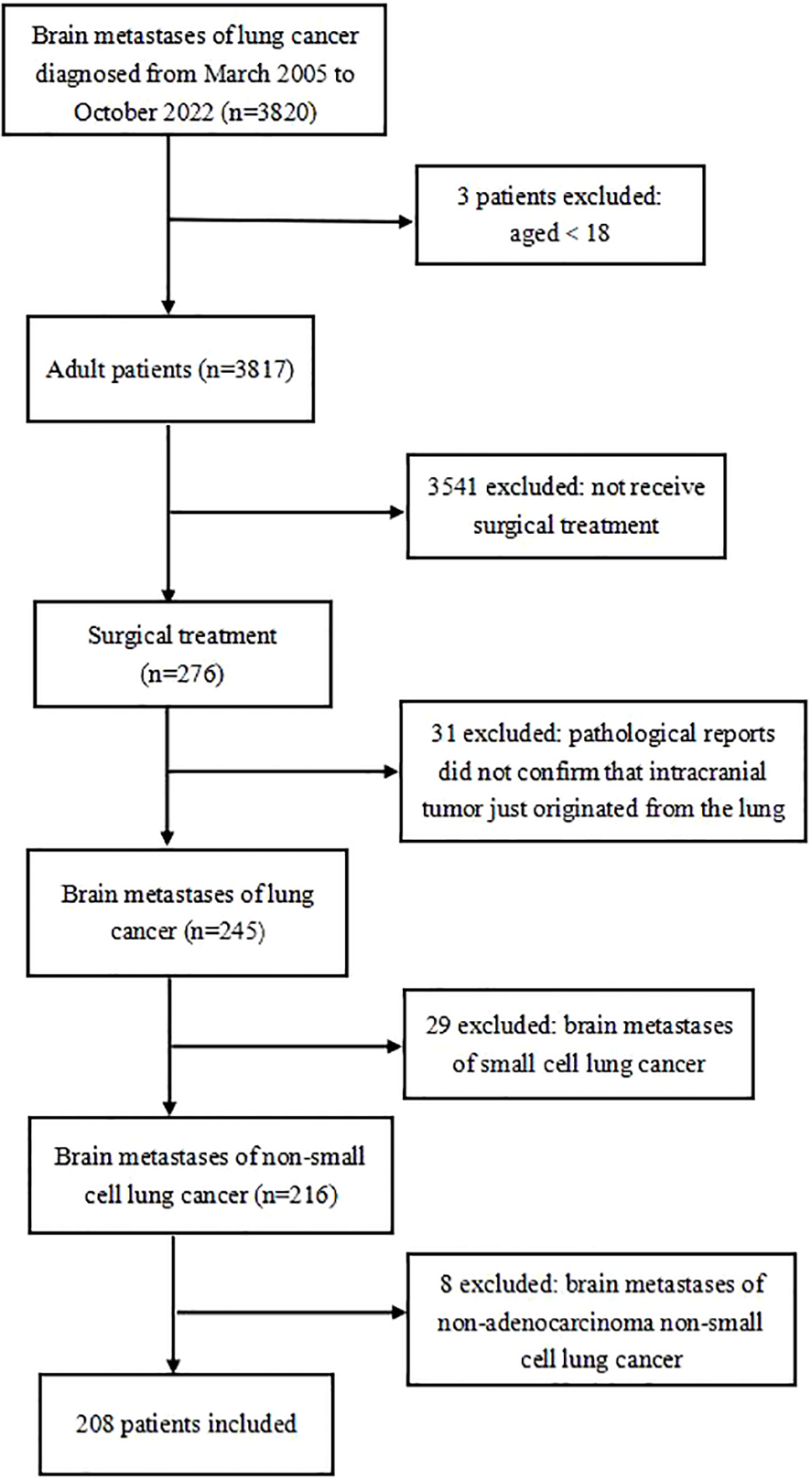
Flow chart of exclusion criteria and study design.

Two hundred eight patients were eventually included in this study (**Figure 1**).

The clinical data on these patients were collected retrospectively from their medical records, including operative and pathological reports, clinical data and information from the office files of their neurosurgeons and thoracic surgeons. Detailed follow-up study of these individuals was conducted by letter and telephone contact with patients, family members, friends, or personal physicians.

This study was conducted by following the Declaration of Helsinki and was approved by the Ethics Committee of Zhengzhou University People’s Hospital, Henan province, China. All patients provided written informed consent before the surgery, and medical records of patients were anonymized.

### 2.2 Driver mutation testing

Genetic mutations were tested through polymerase chain reaction (PCR) or Next-generation sequencing (NGS) using clinical tumor samples. Detections were applied in either plasma DNA or in paraffin-embedded tumor tissue. In the vast majority of patients (75%) in this study, Amplification Refractory Mutation System-qPCR method from AmoyDx, China, was used to detect hotspot mutations in accordance with the manufacturer’s instructions. The panel of PCR included: EGFR, ALK, ROS1, RET, KRAS, NRAS, BARF, HER2, PIK3CA, and MET. In other patients, the Illumia-Nextseq 550 system (Burning Rock, Guangzhou, China) was used to detect driver mutations. It covers 168 cancer genes or 139 cancer genes and can detect structural and copy number changes in addition to hotpot and other coding/splice mutations.

### 2.3 Statistical analysis

All analyses were performed by SPSS 26.0 (IBM Corp., Armonk, NY, USA) software. Descriptive statistics were expressed as numbers and percentages. The end date for the survival analysis for patients lost to follow-up was the time they were last seen in the department or last contacted by telephone. Overall survival was defined as the percentage of patients who were alive at the end of the study period. Survival curves were analyzed using the Kaplan–Meier method with the log-rank test. The univariable and multivariable Cox regression models were conducted to determine the prognostic factors for patients with LUAD BM. Factors with a *P*-value less than 0.05 in the univariable regression analysis were incorporated into the multivariable regression model. All hypothesis tests were conducted against a 2-sided alternative. *P* values were considered statistically significant when less than 0.05.

## 3 Results

### 3.1 Patients characteristics

A total of 208 patients who underwent craniotomy for LUAD BM were identified from 2005 to 2022. The clinical information of this cohort is shown in **Table 1**. Among the patients, 110 patients (52.9%) were males and 98 females (47.1%), and their median age was 61.4 years (23-81years). The majority of patients were farmers (153, 73.6%). In addition, smoking history, alcohol drinking history and the family history of tumor accounted for 40.4% (84 cases), 42.8% (89 cases) and 14.9% (31 cases), respectively. Before craniotomy, only 5 patients (2.4%) had received lung cancer-related treatment at an early stage, of which 2 patients (1.0%) had undergone radical resection of lung cancer, 2 patients (1.0%) had undergone radical resection of lung cancer combined with targeted drug therapy, and 1 patient (0.4%) had only received targeted drug therapy. With respect to symptoms, 203 cases (97.6%) had neurological symptoms, including headache, dizziness, nausea, vomiting, vision loss, slurred speech, limb weakness and seizure. 5 patients (2.4%) had no neurological symptoms, in which PET-CT and physical examination found BM in 3 and 2 patients, respectively.

**Table 1:** Characteristics of our patients who underwent surgical resection for BM of LUAD.

Variables	N (%)
Gender	
Male	110 (52.9)
Female	98 (47.1)
Age	
18-45	19 (9.1)
46-65	142 (68.3)
>65	47(22.6)
Occupation	
Farmer	153 (73.6)
Factory worker	25 (12.0)
Freelancer	11 (5.3)
Emeritus and retired	18 (8.7)
Unemployed personnel	1 (0.4)
Neurological symptoms	
Yes	203 (97.6)
No	5 (2.4)
Smoking history	
Yes	84 (40.4)
No	124 (59.6)
Alcohol drinking history	
Yes	89 (42.8)
No	119 (57.2)
Family history of tumor	
Yes	31 (14.9)
No	177 (85.1)
Treatment before craniotomy	
Yes	5 (2.4)
No	203 (97.6)
The date of the surgery	
2005-2010	14 (6.7)
2011-2015	62 (29.8)
2016-2020	81 (39.0)
2020-2022	51 (24.5)
Location of intracranial metastasis	
Frontal lobe	54 (26.0)
Parietal lobe	20 (9.6)
Temporal lobe	19 (9.1)
Cerebellum	41 (19.7)
Occipital	14 (6.7)
Cranial fossa	6 (2.9)
Other places	16 (7.7)
≥2 or more sites	38 (18.3)
Number of intracranial metastasis	
Single	117 (56.3)
2-4	47 (22.6)
≥5	44 (21.1)
Size of intracranial metastasis (cm)	
0-2	41 (19.7)
2-5	141 (67.8)
>5	26 (12.5)
Gene status	
EGFR	90 (43.3)
ALK	27 (13.0)
KRAS	29 (13.9)
HER2	8 (3.8)
Others	37 (17.8)
No	17 (8.2)
PD-L1 expression	
TPS < 1%	48 (23.1)
1% ≤ TPS ≤ 49%	82 (39.4)
TPS ≥ 50%	78 (37.5)
Ki67	
≤30%	71 (34.1)
>30%	137 (65.9)
CEA	
Positive	71 (34.1)
Negative	50 (24.1)
Unknown	87 (41.8)
S-100	
Positive	71 (34.1)
Negative	112 (53.8)
Unknown	25 (12.1)
KPS after craniotomy	
80-100	63 (30.3)
50-70	80 (38.5)
10-40	65 (31.2)
Post-operative tumor treatment	
No	136 (65.3)
Systemic chemotherapy	30 (14.4)
WBRT	2 (1.0)
Molecular targeted therapy	30 (14.4)
Systemic chemotherapy +WBRT	4 (2.0)
Lung cancer surgery + Systemic chemotherapy	6 (2.9)

Surgery was performed in 14 patients (6.7%) from 2005 to 2010, in 62 patients (29.8%) from 2011 to 2015, in 81 patients (39.0%) from 2016 to 2020, and in 51 patients (24.5%) between 2020 and 2022.

### 3.2 Characteristics of intracranial metastasis

Intracranial metastases were located in the frontal lobe (54, 26.0%), followed by the cerebellum (41, 19.7%), parietal lobe (20, 9.6%), temporal lobe (19, 9.1%), occipital part (14, 6.7%), cranial fossa (6, 2.9%), thalamus (3, 1.4%) and corpus callosum (3, 1.4%), ramus (3, 1.4%), quadrilateral ventricles (2, 1.0%), pineal gland (2, 1.0%), sagittal sinus (2, 1.0%), cingulate gyrus (1, 0.4%). In addition, 38 patients (18.3%) had intracranial metastases in 2 or more sites.

With respect to the number of intracranial metastases, 117 patients (56.3%) had a solitary BM and 91 (43.7%) had multiple metastatic lesions. The tumors were divided into 3 grades according to their maximum diameters. 41 cases (19.7%) had a maximum diameter of 0 to 2 cm, 141 cases (67.8%) had a maximum diameter of 2 to 5 cm, and 26 cases (12.5%) had a maximum diameter of more than 5 cm.

Nearly half of the patients (90, 43.3%) had EGFR mutations; 29 cases (13.9%) had KRAS mutations, 27 cases (13.0%) ALK rearrangement, 8 cases (3.8%) Her2 mutations, and 37 cases (17.8%) other mutation. In addition, 17 cases (8.2%) had non-actionable mutations.

One of the commonly used methods for assessing PD-L1 expression is the Tumor Proportion Score (TPS), which considers the percentage of tumor cells expressing PD-L1. PD-L1 status was categorized into positive (TPS ≥1%) and negative (TPS <1%) expression. Immunohistochemical results of BM showed that PD-L1 was positive in 160 patients (76.9%) and negative in 48 patients (23.1%). There were 137 patients (65.9%) with Ki67 > 30%, and 71 patients (34.1%) with Ki67 ≤ 30%. S-100 was positive in 71 patients (34.1%) and negative in 112 patients (53.8%), and the result was missing in 25 patients (12.1%).

### 3.3 Karnofsky performance score after craniotomy

Patients enrolled had Karnofsky Performance Score (KPS) assessed one week post-discharge. KPS was categorized into low (10-40), intermediate (50-70) and high (80-100). The mean ± standard deviation of KPS in the entire cohort was 58 ± 24. Low, intermediate and high performance status were seen in 65 cases (31.2%), 80 cases (38.5%), and 63 cases (30.3%) of the cohort, respectively.

### 3.4 Treatment characteristics

All 208 (100%) patients underwent surgery for LUAD BM but only 72 (34.7%) continued additional tumor treatment after craniotomy. In these cases, 30 patients (14.4%) underwent postoperative chemotherapy (pemetrexed and cisplatin based), 2 patients (1.0%) underwent WBRT, 30 patients (14.4%) underwent molecular targeted therapy (28 patients receiving EGFR tyrosine kinase inhibitors (TKIs) and 2 patients ALK TKIs), 4 patients (2.0%) received chemotherapy combined with WBRT, 6 patients (2.9%) underwent lung cancer resection combined with chemotherapy. No patients received post-operative immunotherapy.

### 3.5 Survival outcomes

**Figure 2:**
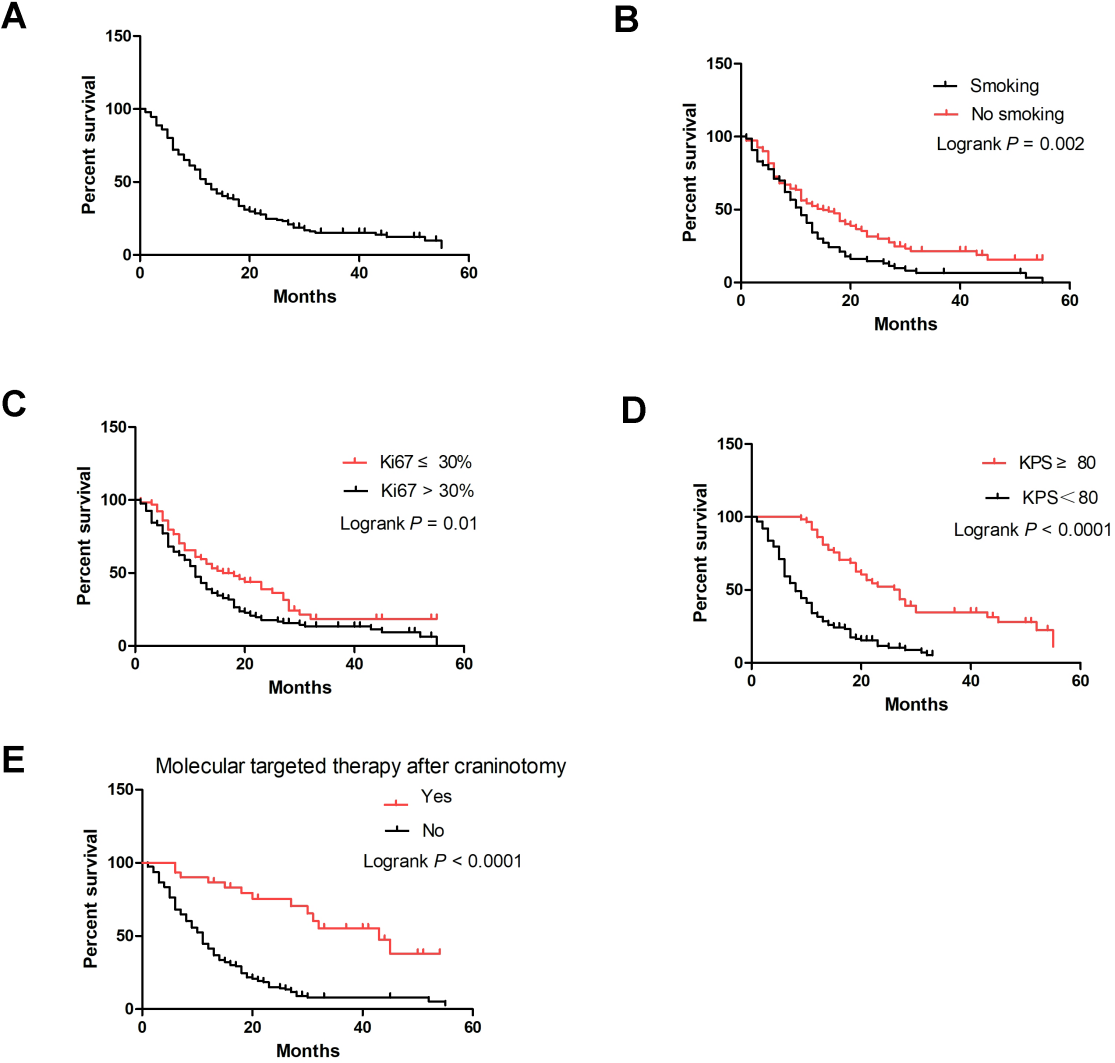
Kaplan–Meier curve of LUAD brain metastases patients undergoing surgical resection. **(A)** Overall survival curve of 190 patients; **(B)** Smoking-specific survival curves of patients; **(C)** Ki67 percentage–specific survival curves; **(D)** KPS-specific survival curves; **(E)** Molecular targeted therapy after craniotomy-specific survival curves.

A total of 190 patients (90.7%) were followed up successfully. Among these patients, survival time ranged from 0 to 55 months, with the median survival time being 11.5 months. The 3-month, 6-month, 1-year, 3-year and 4-year survival rates were 88.77%, 72.09%, 48.78%, 15.17% and 12.64%, respectively (**Figure 2A**). In the univariate Cox analysis, smoking history, Ki67 percentage, KPS after craniotomy, and molecular targeted therapy after craniotomy were significantly associated with overall survival (OS) (*P*<0.05) (**Table 2**). **Figures 2B-E** shows the Kaplan–Meier survival plots generated from curves stratified according to the smoking history, Ki67 percentage, KPS after craniotomy, and molecular targeted therapy after craniotomy. Gender, age, neurological symptoms, alcohol drinking history, history of lung cancer treatment, the number of intracranial metastases, size of metastases, PD-L1 expression, EGFR mutation, and systemic chemotherapy after craniotomy did not significantly affect the survival. Multivariate analysis revealed that smoking history (HR=0.677, P=0.02), Ki67 percentage (HR=0.594, P=0.004), KPS after craniotomy (HR=0.279, P=0.000) and molecular targeted therapy after craniotomy (HR=3.589, *P*=0.000) were independent factors affecting the survival time of patients (**Table 2**).

**Table 2:** Univariate and multivariate Cox proportional hazard models for overall survival in 190 cases who had surgical resection for BM of LUAD.

Variables	n	Univariate analysis		Multivariate analysis	
		HR (95%CI)	*P*	HR (95% CI)	*P*
Gender					
F/M	88/102	1.338 (0.969-1.846)	0.077		
Age					
18-65/≥66	146/44	0.854 (0.58-1.257)	0.422		
Neurological symptoms					
No/Yes	5/185	0.639 (0.203-2.007)	0.443		
Smoking history					
No/Yes	113/77	0.641 (0.465-0.883)	0.007	0.677 (0.487-0.94)	0.02
Alcohol drinking history					
No/Yes	110/80	1.194 (0.864-1.649)	0.282		
History of lung cancer treatment					
No/Yes	185/5	3.567 (0.881-14.443)	0.075		
Number of intracranial metastasis					
1/≥2	105/85	1.03(0.747-1.42)	0.858		
≤4/>4	149/41	1.017(0.687-1.504)	0.933		
Size of intracranial metastasis					
≤5cm/>5cm	164/26	0.893(0.576-1.384)	0.611		
Ki67 positive					
≤30%/>30%	65/125	0.629 (0.444-0.892)	0.009	0.594 (0.417-0.847)	0.004
EGFR mutation					
Yes/No	81/109	1.034(0.749-1.427)	0.838		
PD-L1 expression					
<1%/≥1%	45/145	0.921 (0.628-1.35)	0.672		
KPS after craniotomy					
≥80/<80	58/132	0.279 (0.188-0.414)	0.000	0.279 (0.187-0.417)	0.000
Systemic chemotherapy after craniotomy					
No/Yes	160/30	1.451 (0.929-2.265)	0.102		
Molecular targeted therapy after craniotomy					
No/Yes	160/30	4.442 (2.488-7.930)	0.000	3.589 (1.996-6.453)	0.000

## 4 Discussion

Lung cancer ranks first in terms of morbidity and mortality among all tumors worldwide, and the brain is a common metastasis site of lung cancer (15, 16). The incidence of BM in patients with lung cancer is approximately 20%-65%, which seriously affects the prognosis and quality of life (QOL) of patients (3, 4). Despite treatment, the majority of patients still die due to progressive BM. Although prognosis in patients with BM from lung cancer is usually poor, prolonged survival can be observed in a subgroup of patients with favorable prognostic factors (8, 9). In these favorable subgroups, more aggressive treatment modalities can be used to increase QOL and influence survival. BM are a complex condition with multiple factors contributing to diagnosis and prognosis. The blood-brain barrier has limited the effective penetration of many systemic therapies into the brain. Surgical option is feasible as an emergency strategy and also suggested as an elective procedure to improve neurological status, QOL, and survival.

In this study, the clinical data of 208 patients with LUAD BM undergoing surgical resection were retrospectively collected and analyzed. Patients age ranged from 23 to 81 years old, among which 46-65 years old (142 cases, 68.3%) accounted for a large proportion of patients. COX regression model found that age was not a factor affecting the prognosis of patients. Similarly, in Shen’s study, they analyzed the Surveillance, Epidemiology, and End Result (SEER) database and identified that age was not an independent prognostic factor of lung cancer BM (17). Other studies have reported that the incidence of LUAD BM increases with age, and this phenomenon may be related to living standards and physical conditions (18, 19). However, since the population included in this study was LUAD BM who underwent craniotomy, some patients who were old and frail and whose overall physical condition was not suitable for surgery were excluded. Evaluation of age on the prognosis of LUAD BM who underwent craniotomy requires a larger sample size. In addition, people aged 46-65 years old are the major society contributors, and attention should be paid to the characteristics of people in this stage to achieve early detection and early treatment.

Cigarette use is the major cause of morbidity and mortality in developed countries, increasing all cause mortality (20, 21). Smoking intervention could reduce the frequency of postoperative complications and improve the prognosis of lung cancer patients (22–24). However, there is limited data on the effects of smoking on LUAD BM. In our study, we found that smoking is an important factor affecting the prognosis of patients with LUAD BM; the survival time of smokers was significantly shorter than that of non-smokers. We speculate that this reason may be that tobacco smoke contains hundreds of known and probable human carcinogens (25), which accelerate the metastasis of cancer cells and aggravate the nervous system damage in LUAD BM patients. Shenker has reported that in patients with BM from lung cancer who received SRS, current smoking status and pack-year history of smoking had no effect on overall survival, however, current smokers with non-adenocarcinoma lung cancers had a trend toward greater neurologic death than nonsmokers and cumulative pack years smoking is associated with a greater BM velocity (26). These results indicated the dangers of smoking and the need for smoking cessation in the general population as well as in patients with BM from LUAD.

Ki67 is a well-known marker for the evaluation of cell proliferation. Numerous studies have indicated that Ki67 index independently predicts cancer progression. Immunostaining for Ki67 expression is the gold standard, and the higher positivity of Ki67 expression is, the stronger invasion ability of tumor cells (27). Berghoff et al. (28) found a correlation between Ki67 and survival time; high Ki67 expression showed a shorter survival time. In line with these findings, in our study, the prognosis of LUAD patients with BM with high expression of Ki67 was poor, and Ki67 was an independent factor affecting the prognosis of patients.

KPS has been used broadly in clinical practice to assess the overall performance status of cancer patients’ activity, work, and self-care abilities (29). A multicenter, retrospective cohort study showed that neurosurgical resection improved OS and was associated with a significantly better prognosis in patients with lung cancer BM and poor KPS (30). We assessed LUAD BM KPS one week post-discharge and found that patients with higher KPS had a better prognosis, which indicated that patients with preserved functional capacity benefit from craniotomy over those with lower KPS.

Our study cohort is unique since the overwhelming majority (97.6%) of the patients (203 patients) were admitted to the neurosurgery department due to neurological symptoms, without prior diagnosis of lung cancer. Three (1.4%) of the other 5 patients (2.4%) were diagnosed with lung cancer after relevant examination, which suggests the occult occurrence of lung cancer and the importance of early screening. Among the 208 patients, 5 patients (2.4%) had received lung cancer-related treatment at an early stage, including 2 patients (0.9%) with radical resection of lung cancer, 2 (0.9%) with radical resection of lung cancer combined with targeted drug therapy, and 1 (0.45%) with molecular targeted therapy. The Univariate COX model found that whether the patients underwent lung cancer-related treatment before BM surgery was not a factor affecting the postoperative survival time of patients with LUAD BM. The reason may be related to the small number of patients who underwent lung cancer-related treatment before BM surgery (just 5 cases), and the sample size will be expanded in future analysis.

The incidence of brain metastasis has increased in parallel with LUAD incidence, accuracy of imaging examination and people’s attention to physical examination. The role of surgery in the management of LUAD BM is indisputable. Surgery can quickly alleviate life-threatening symptoms caused by tumor effects, cerebrospinal fluid obstruction, and edema around the tumor. Surgical resection of BM is typically indicated for solitary intracranial metastasis (31, 32). Surgical treatment was not considered for patients with multiple metastases (more than 3 locations) that were considered to have uncontrolled primary tumors and poor prognosis (33). However, rapid advances in systemic therapy are improving outcomes for this patient population across multiple tumor types, generating new interest in local control strategies (34). Sauvageot et al. published a retrospective cohort study of 184 patients with 72 patients (39.1%) having more than 2 BM. The median OS was 19.2 months and the median cerebral progression-free survival was 8.4 months. The number of BM and tumor cerebral burden remained significant prognostic factors for OS (35). Pollock et al. published a retrospective cohort study of 52 patients with a median of three BM. The result showed that 5 patients who received resection alone survived a median of 19 months; 31 patients who received radiosurgery alone survived a median of 13 months and 16 patients who received both resection and radiosurgery survived a median of 8 months (36). Based on these results, resection was recommended for patients with multiple BM. In our study, 72.1% of the patients survived for more than 6 months, and 15.2% survived for more than 3 years. This survival outcome was better than the survival time expected for LUAD BM, suggesting that resection should be considered as an option, even in the case of patients with multiple BM, in order to improve their survival and QOL.

In addition, of the 190 patients (91.3%) who were successfully followed up, only 72 patients (34.7%) received post-operative treatment. The use of biomarker-matched target therapies has been considered solely responsible for improving population-level lung cancer-specific mortality between 2013 and 2016 (37). In 2 published retrospective studies of NSCLC patients with BM and EGFR mutations, patients who received an EGFR TKI at any time after diagnosis of BM survived longer than those who did not (38, 39). Both the Gow et al. and Eichler et al. series suggested that EGFR TKI therapy after the initial diagnosis of BM may provide such a substantial benefit in duration of central nervous system response that survival is ultimately impacted. Similarly, in our study, patients who received molecular targeted therapy after craniotomy showed improved overall survival compared with patients who did not, and multivariate analysis revealed molecular targeted therapy after craniotomy as an independent factor affecting the survival time of patients. Taken together, these data suggest that LUAD patients with BM could benefit from molecular targeted therapy after BM resection.

The reasons for most patients not receiving post-operative treatment were as follows: first, some patients could not tolerate the systemic treatment because of the treatment side effects; second poor KPS after surgery; and third economic considerations. In addition, when the patients knew that the pathological diagnosis was malignant tumor, most of them chose to give up the postoperative treatment.

Our study has some limitations. First, it is a retrospective study at a single center and the sample size is limited, which may have led to a bias in patient selection and limited firm conclusions. Therefore, our results may not apply to other populations and other centers. Further multicenter prospective studies are thus recommended. Second, the clinical data is limited; we were unable to collect hematology examination and cerebrospinal fluid examination, which may be important factors affecting the survival time of patients. Third, the study was focused on LUAD BM who underwent craniotomy at our center, and most patients did not receive treatment post-operatively. No patients received SRS which is a recommendation of NCCN for low tumor volume, both to resection cavity and any other non-resected BM. Our data did not fully represent the overall status of all lung cancer patients with BM. Despite these limitations, to the best of our knowledge, it is the largest published cohort that clarifies the clinical characteristics and prognosis factors of LUAD BM treated with surgical resection, which may be relevant to similar patient cohorts. In the follow-up study, we will carry out a multi-center study, further expand the sample size, and improve the clinical data by incorporating tumor genetics, hematology and cerebrospinal fluid to determine the factors affecting the survival of patients undergoing craniotomy. Meanwhile, we will collect all the data of patients with lung cancer BM as much as possible to present the comprehensive status of the patients in an effort to improve the prognosis of patients.

## 5 Conclusion

In summary, our study demonstrates the clinical characteristics and prognostic factors of patients undergoing craniotomy for LUAD BM. The intracranial location of BM was mostly in the frontal lobe and the metastatic lesions were mostly single; the metastatic tumor size was mostly between 2-5 cm. Moreover, nearly half of the patients had EGFR mutations and most had Ki67 > 30% and positive PD-L1. Few patients received lung tumor treatment before craniotomy, and only a minor subset continued tumor therapy after craniotomy. Although the 3-year survival was poor, surgical resection for patients who had no-smoking history, Ki67 percentage ≤30%, KPS≥80 after craniotomy, and molecular targeted therapy after craniotomy can improve the prognosis and prolong the survival time.

## Data Availability

The original contributions presented in the study are included in the article/supplementary material. Further inquiries can be directed to the corresponding authors.
